# Описание клинических случаев нарушений формирования пола 46,XY, обусловленных мутацией в гене <i>DHH</i>. Роль сигнального пути DHH в формировании пола

**DOI:** 10.14341/probl12757

**Published:** 2021-06-07

**Authors:** Н. Ю. Калинченко, З. К. Батырова, И. Б. Кострова, А. А. Колодкина, Е. В. Уварова, З. Х. Кумыкова, А. В. Асатурова, Г. Н. Хабас, А. Н. Тюльпаков

**Affiliations:** Национальный медицинский исследовательский центр эндокринологии; Национальный медицинский исследовательский центр акушерства, гинекологии и перинатологии им. академика В.И. Кулакова; Детская республиканская клиническая больница им. Н.М. Кураева; Национальный медицинский исследовательский центр эндокринологии; Национальный медицинский исследовательский центр акушерства, гинекологии и перинатологии им. академика В.И. Кулакова; Национальный медицинский исследовательский центр акушерства, гинекологии и перинатологии им. академика В.И. Кулакова; Национальный медицинский исследовательский центр акушерства, гинекологии и перинатологии им. академика В.И. Кулакова; Национальный медицинский исследовательский центр акушерства, гинекологии и перинатологии им. академика В.И. Кулакова; Национальный медицинский исследовательский центр акушерства, гинекологии и перинатологии им. академика В.И. Кулакова; Национальный медицинский исследовательский центр эндокринологии; Медико-генетический научный центр им. акад. Н.П. Бочкова; Республиканская детская клиническая больница

**Keywords:** нарушение формирования пола, дисгенезия гонад, <i>DHH</i>, первичная аменорея, периферическая минифасцикулярная полинейропатия, секвенирование нового поколения

## Abstract

Мутации в гене DHH являются крайне редкой причиной возникновения нарушений формирования пола (НФП) 46,XY. В статье описываются клинические наблюдения двух неродственных пациенток с дисгенезией гонад 46,XY и правильным женским фенотипом. Благодаря применению метода секвенирования нового поколения (NGS) у обеих выявлена одинаковая биаллельная вариантная замена c.419T>G в гене DHH. Учитывая данные литературы о роли DHH в формировании нервной системы, установление генетической причины заболевания позволило в обоих случаях диагностировать минифасцикулярную полинейропатию на доклиническом этапе. Таким образом, приведенные нами клинические случаи демонстрируют ценность использования NGS, позволяющего одновременно анализировать широкий спектр генов-кандидатов при НФП и диагностировать сопутствующие заболевания до развития клинической картины. Это первые описания пациентов с мутациями в гене DHH в российской популяции.

## Abstract (en)

Mutations in the gene DHH are an extremely rare cause of disorders of sex development 46,XY (DSD,46XY). The article describes the clinical cases of two unrelated patients with gonadal dysgenesis 46,XY with female phenotype. By using a next generation sequencing method, in both cases the same biallelic variant substitution c. 419T>G in the DHH gene was revealed. Taking into account the data on the role of DHH in the formation of the nervous system, the diagnosis of minifascicular polyneuropathy at the preclinical stage was confirmed in both cases. These cases demonstrate the value of using NGS, which allows simultaneous analysis of a wide range of candidate genes in DSD and the diagnosis of comorbidities before the development of the clinical picture. These are the first descriptions of patients with mutations in the DHH gene in the Russian population.

## АКТУАЛЬНОСТЬ

Нарушения формирования пола (НФП) 46,XY — гетерогенная группа состояний, при которых наблюдается несоответствие строения наружных и/или внутренних половых органов хромосомному мужскому полу 46,XY. Одной из причин возникновения НФП 46,XY является нарушение закладки гонад или их дифференцировки (дисгенезия гонад — ДГ).

Началом к активному изучению механизмов возникновения ДГ послужило открытие в 1990 г. гена SRY, картированного на Y-хромосоме, как ключевого фактора развития яичка из бипотенциальной гонады [1]. Позже было показано, что SRY запускает каскад активации транскрипционных факторов, участвующих в развитии яичка, таких как SOX9, GATA4, ZFPM2, NR5A1, WT1, DHH, CBX2, ATRX, MAP3K1, FGF9 и другие, нарушения функции которых также будут приводить к ДГ.

Учитывая редкость данных состояний, описание каждого случая ДГ является уникальным, т.к. изучение нарушений функционирования данных транскрипционных белков, приводящих к ДГ 46,XY, помогает понять механизмы формирования пола и объяснить значительную фенотипическую вариабельность среди данной группы пациентов. Ниже мы приводим первые в России клинические описания ДГ 46,XY, обусловленные мутацией в гене DHH.

## МАТЕРИАЛЫ И МЕТОДЫ

Пациентам проводилось комплексное обследование, включающее: оценку строения наружных половых органов, ультразвуковое исследование (УЗИ) малого таза, паховых каналов, исследование гормонального статуса — уровней лютеинизирующего, фолликулостимулирующего, антимюллерова гормонов (ЛГ, ФСГ, АМГ), тестостерона, эстрадиола, проведение электромиографии.

Молекулярно-генетический анализ проводился в лаборатории отделения наследственных эндокринопатий ФГБУ «НМИЦ эндокринологии» Минздрава России. Геномную ДНК выделяли из лейкоцитов периферический крови стандартным методом (набор Pure Link, Genomic DNA Mini Kit, Life Technologies, США). Для молекулярно-генетического анализа применялся метод секвенирования нового поколения — NGS (Next Generation Sequencing). Использовалась разработанная в отделении наследственных эндокринопатий ФГБУ «НМИЦ эндокринологии» Минздрава России панель праймеров для мультиплексной полимеразной цепной реакции (ПЦР) и секвенирования с применением технологии Ion Ampliseq™ Custom DNA Panel (Life Technologies, США). Панель праймеров «Нарушения формирования пола» охватывает кодирующие области следующих генов: AKR1C2, AKR1C4, AMH, AMHR2, AR, ARX, ATRX, CBX2, CYB5A, CYP11A1, CYP17A1, DHCR7, DHH, EMX2, ESR2, FGD1, FGF9, FGFR2, FKBP4, FOXF2, FOXL2, HOXA13, HSD17B3, HSD3B2, ICK, LHCGR, LHX1, LHX9, MAMLD1, MAP3K1, MID1, NR0B1, NR5A1, POR, PTGDS, SOX9, SRD5A2, SRY, STAR, SUPT3H, TSPYL1, WNT4, WT1, ZFPM2. Подготовка библиотек проводилась в соответствии с рекомендациями производителей. Секвенирование осуществлялось на полупроводниковом секвенаторе PGM (Ion Torrent, Thermo Scientific, США) или Illumina MiSeq (Illumina, США). Биоинформатическая обработка результатов секвенирования проводилась с помощью программных модулей Torrent Suite 4.2.1 (Ion Torrent, Life Technologies, США) или Genome Analysis ToolKit (GATK) ver. 4.1.2.0 (Broad Institute, Cambridge, MA, USA). Для аннотирования вариантов нуклеотидной последовательности использовался пакет программ ANNOVAR ver. 2018Apr16. После анализа полученных данных проводилось подтверждение полученных мутаций на секвенаторе Genetic Analyzer Model 3130 (Life Technologies, США). Оценка патогенности вариантов нуклеотидной последовательности проводилась согласно международным и российским рекомендациям [2][3]. Нумерация кодирующей последовательности гена DHH дана по референсу NM_021044 (http://www.ncbi.nlm.nih.gov/ genbank).

Клиническое наблюдение №1.

Больная У., 14 лет обратилась в связи с задержкой развития вторичных половых признаков и отсутствием визуализации внутренних половых органов по данным инструментальных исследований (УЗИ малого таза и магнитно-резонансная томография (МРТ) малого таза).

Из анамнеза известно, что родители девочки — аварцы, близкородственный брак отрицают. Девочка — единственный ребенок в семье.

При физикальном осмотре половое развитие соответствовало стадии 1 по Таннеру (P1B1). Наружные половые органы сформированы по женскому типу, уретра расположена типично, гимен бахромчатый. Влагалище заканчивается слепо, глубиной до 3,5 см. При ректальном осмотре: тело матки и придатки не пальпируются.

По данным гормонального профиля выявлен гипергонадотропный гипогонадизм: ЛГ — 77,8 МЕ/л (5,5–9,4), ФСГ — 170 МЕ/л (5,1–9,8), эстрадиол — <73,4 пмоль/л (261–394), АМГ — 2,4 нмоль/л (1–10,6). Остальные гормональные показатели были в пределах нормативных значений. Определен кариотип 46,XY.

По данным МРТ матка и яичники в типичном месте не визуализированы, а в паховых каналах с обеих сторон обнаружены гонады (тестикулы?).

Установлен клинический диагноз: Нарушение формирования пола, 46,XY. Выполнены лапароскопия, двустороннее удаление дисгенетичных гонад.

По данным гистологического исследования: гонады с обеих сторон представлены тканью яичка, в которой все трубочки замещены клетками Сертоли, придаток яичка справа обычного строения, в тазовых смывах группы клеток мезотелия (рис. 1).

**Figure:**
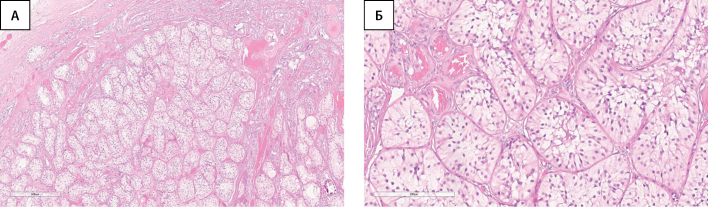
Рисунок 1. Ткань яичка (без признаков неопластических изменений).А — окраска гематоксилином и эозином, х40. Б. — окраска гематоксилином и эозином, х200.

Для уточнения генеза заболевания проведено NGS, по результатам которого в гене DHH выявлен гомозиготный вариант c.419T>G:p.L140R, ранее не описанный, вероятно патогенный.

Учитывая данные литературы [4] о сочетании НФП с минифасцикулярной полинейропатией (МФПНП) при мутациях в DHH, несмотря на отсутствие клинических проявлений нейропатии, проведена электронейромиография (ЭНМГ), по данным которой выявлены признаки генерализованного симметричного умеренно выраженного аксонально-демиелинизирующего поражения моторных и сенсорных волокон периферических нервов верхних и нижних конечностей. Изменения более выражены в нижних конечностях (дистальные моторные и сенсорные ответы получены не были).

Девочке назначена заместительная гормональная терапия и рекомендовано наблюдение и ведение неврологом.

Клиническое наблюдение №2.

Больная Ч., фенотипическая девочка, впервые обратилась с жалобами на задержку полового развития в возрасте 14 лет. Из анамнеза известно, что родители пациентки аварцы по национальности и являются родственниками во втором поколении. При осмотре телосложение пропорциональное, стигмы дисэмбриогенеза отсутствовали, половой статус по Таннеру: В1, P2, половые органы сформированы правильно, по женскому типу. В гормональном профиле выявлено значительное повышение гонадотропинов: ЛГ — 21 МЕ/л (0,03–3,9), ФСГ — 74 МЕ/л (0,68–6,7), на фоне низко-нормального уровня эстрадиола — 41,3 пмоль/л (40–240), АМГ — 0,1 нмоль/л (1,6–10,0), все другие тропные и стероидные гормоны определялись в пределах референсных значений. При УЗИ и МРТ органов малого таза: гонады ни в малом тазу, ни в паховых каналах не определялись, матка не визуализировалась, определялась дистальная часть влагалища. Определен кариотип 46,XY.

На основании проведенного обследования установлен диагноз «Нарушение формирования пола 46,XY, чистая ДГ». Пациентке проведена диагностическая лапароскопия, в ходе которой удалены маточные трубы с обеих сторон, дисгенетичные гонады. По данным гистологического исследования: «две гонады представлены примитивными семенными канатиками, функциональная активность отсутствует, герминативные клетки не просматриваются, данных за онкологический процесс не получено».

Для уточнения генеза заболевания проведено NGS, по результатам которого в гене DHH выявлен гомозиготный вариант c.419T>G:p.L140R, ранее не описанный, вероятно патогенный. Родители пациентки оказались гетерозиготными носителями выявленной мутации.

Пациентке проведена ЭНМГ, в ходе которой выявлены изменения, аналогичные случаю 1: признаки генерализованного симметричного умеренно выраженного аксонально-демиелинизирующего поражения моторных и сенсорных волокон периферических нервов верхних и нижних конечностей, выраженное сенсорное нарушение срединных, икроножных и правого глубокого малоберцового нервов аксонального характера, подтверждающее наличие генерализованной нейропатии. Таким образом, несмотря на отсутствие каких-либо жалоб, ассоциированных с нейропатией, у пациентки на доклиническом уровне установлено наличие МФПНП. Рекомендовано наблюдение невропатологом.

## ОБСУЖДЕНИЕ

Высококонсервативное семейство белков Hedgehog — это группа сигнальных молекул, играющих центральную роль в развитии и регуляции процессов эмбрио- и морфогенеза. У млекопитающих обнаружены три гомолога полипептида Hedgehog: Desert (Dhh), Indian (Ihh) и Sonic (Shh). Shh участвует в формировании конечностей, закладке нервной трубки. Ihh регулирует развитие хондроцитов, формирование скелета. Dhh играет ключевую роль в развитии гонад. Показано, что у мышей Dhh начинает продуцироваться клетками Сертоли с 11,5 дней внутриутробного развития, через короткий промежуток времени после инициации экспрессии Sry (sex-determining region of the Y chromosome) [5]. SRY индуцирует активацию транскрипционных факторов, определяющих специализацию клеток бипотенциальной гонады в сторону яичка, в том числе появление первых, специфичных для яичка функционально активных клеток Сертоли. Секретируемый клетками Сертоли DHH связывается с рецептором PTCH1, расположенным на предшественниках фетальных клеток Лейдига, запуская сигнальный путь hedgehog, который приводит к специализации стероидогенных клеток и активации экспрессии гена NR5A1 (кодирующего стероидогенный фактор 1 — SF1). Последний является ключевым регулятором стероидогенеза в гонадах. Также DHH регулирует дифференцировку перитубулярных миоидных клеток и формирование семенных канатиков [6][7]. В эксперименте на мышах показано, что самцы с нокаутом гена Dhh -/- имеют женский фенотип и слепо заканчивающееся влагалище, тогда как самки Dhh -/- фертильны [8]. Данное исследование демонстрирует эксклюзивную роль Dhh в дифференцировке яичка и отсутствие его влияния на формирование женской репродуктивной системы. Интересно, что, помимо важной роли в развитии мужской репродуктивной системы, Dhh принимает участие в формировании периневральной трубки и в развитии оболочек периферической нервной системы у лиц обоих полов.

У человека ген DHH (12q12-q13) состоит из 3 экзонов и кодирует белок из 396 аминокислотных остатков [9]. ДГ 46,XY вследствие патологических вариантных замен в гене DHH является редким аутосомно-рецессивным заболеванием. К настоящему времени в литературе описано не более 20 случаев. Фенотип описанных пациентов варьировал от правильного женского строения наружных половых органов до мошоночной формы гипоспадии, в большинстве случаев дериваты мюллеровых протоков отсутствовали. В 5 случаях в удаленных гонадах по данным гистологии имелись признаки малигнизации [10][11].

Впервые в 1999 г. Umehara F. и соавт. [4] описали 46-летнюю женщину со смешанной формой ДГ 46,XY в сочетании с МФПНП, обусловленной гомозиготной миссенс-мутацией в гене DHH. Позже были описаны пациенты без МФПНП и с различными вариантами ДГ (чистая ДГ, смешанная ДГ). Среди описанных случаев патологические замены в гене DHH располагались во всех трех экзонах, без какой-либо преимущественной локализации [10–12].

Исключительная роль DHH у человека в дифференцировке бипотенциальной гонады только при кариотипе 46,XY продемонстрирована Baldinotti F. и соавт. [13] при описании семейного варианта гомозиготной мутации c.554C>A, приводящей к стоп-кодону p.Ser185* в гене DHH. При наличии аналогичных гомозиготных мутаций у двух сестер с кариотипами 46,XY и 46,XX и подтвержденной у обеих МФПНП ДГ диагностирована только у сестры c кариотипом 46,XY, тогда как у сестры с кариотипом 46,XX нарушения репродуктивной функции отсутствовали.

В ряде последних научных публикаций обсуждается роль гетерозиготных вариантных замен в гене DHH при наличии у мужчин изолированной азооспремии, двустороннего крипторхизма или гипоспадии. Так, Mehta и соавт. [14] при анализе 125 нефертильных мужчин с астенозооспермией обнаружили в 6,6% случаев синонимичную замену c. 543C>T (p.His181His) в гене DHH и вставку G c.1156insG (p.Arg385fs) у пациента с двусторонним крипторхизмом и азооспермией. В работе Ayers и соавт. [10] описаны гетерозиготные замены в гене DHH среди пациентов с гипоспадией. Авторами предполагается наличие у данных пациентов дополнительных вариантов в интронных областях (что будет укладываться в рецессивный характер заболевания) либо наличие вариабельной пенетрантности или дополнительных неустановленных изменений в других генах, участвующих в сигнальном пути DHH [10].

В представленных нами описаниях 2 пациентов с женским фенотипом при кариотипе 46,XY выявлен ранее не описанный биаллельный вариант c.419T>G, приводящий к замене неполярной гидрофобной аминокислоты лейцина на полярную гидрофильную аминокислоту аргинин (p.L140R). Данная мутация определена как вероятно патогенная [2][3]. Более того, у обоих пациентов установлено наличие МФПНП по данным ЭМНГ, что косвенно подтверждает нарушения в функции DHH у наших пациентов.

Факт обнаружения одинаковой гомозиготной мутации у двух пациенток из разных семей, в которых оба родителя аварцы, позволяет предполагать наличие «эффекта основателя» для данной вариантной замены. Полученные данные позволят проводить медико-генетическое консультирование при планировании семьи и оценке рисков рождения детей с ДГ вследствие патологической вариантной замены в гене DHH.

## ЗАКЛЮЧЕНИЕ

Таким образом, впервые в России нами представлено описание случаев НФП 46,XY, обусловленных биаллельной мутацией в гене DHH. Данные наблюдения подчеркивают диагностическую ценность NGS, позволяющего одновременно анализировать широкий спектр генов-кандидатов при НФП, а также расширяют наши представления о механизмах дифференцировки тестикулярной ткани и патогенезе ДГ.
